# 40 Years without Smallpox

**Published:** 2017

**Authors:** G. A. Shchelkunova, S. N. Shchelkunov

**Affiliations:** State Research Center of Virology and Biotechnology VECTOR, Koltsovo, Novosibirsk region, 630559 , Russia; Novosibirsk State University, Pirogov Str. 2, Novosibirsk, 630090, Russia

**Keywords:** smallpox, variola (smallpox) virus, evolution, DNA diagnostics, vaccine, chemotherapeutic drugs

## Abstract

The last case of natural smallpox was recorded in October, 1977. It took humanity almost 20 years to achieve that feat after the World Health Organization had approved the global smallpox eradication program. Vaccination against smallpox was abolished, and, during the past 40 years, the human population has managed to lose immunity not only to smallpox, but to other zoonotic orthopoxvirus infections as well. As a result, multiple outbreaks of orthopoxvirus infections in humans in several continents have been reported over the past decades. The threat of smallpox reemergence as a result of evolutionary transformations of these zoonotic orthopoxviruses exists. Modern techniques for the diagnostics, prevention, and therapy of smallpox and other orthopoxvirus infections are being developed today.

## INTRODUCTION

Smallpox (also known by its Latin name *variola vera, *derived from *varius *(spotted) or *varus *(pimple)) got its current name as early as the 16^th^ century, although the disease had been known since ancient times and had inflicted a heavier toll on humans than many other infections and numerous wars. In the 20^th^ century alone, over the almost 80 years during which mass vaccination against smallpox and an intensive anti-epidemic campaign were being conducted, 300 million people still died as a result of the disease [1].

In 1796, the English physician Edward Jenner proposed a method for protection against smallpox via the inoculation of the infectious material obtained from cows with a smallpox-like disease. This method became known as *vaccination *(from the Latin *vacca *for cow). This breakthrough event took place almost a century before the kingdom of viruses was discovered [1-3].

After its introduction in 1919, mandatory smallpox vaccination in Russia (and then, in the Soviet Union), a vast country with manifold geographic conditions ranging from high-mountain expanses and deserts to northern tundra and outlandish taiga areas and home to dozens of nationalities differing in traditions, rites, and religious practices, made it possible to eliminate smallpox morbidity by 1936 [3].

This dangerous, highly contagious disease was eradicated in many developed countries in the first half of the 20^th^ century. However, smallpox outbreaks were still recorded each year in 50–80 countries even in the 1950s. In addition, the foci of endemic smallpox in Asia, Africa, and South America posed a constant threat of importation to countries already free of the disease.

Based on an analysis of the tremendous scientific and organizational expertise on smallpox eradication accumulated in the Soviet Union, V.M. Zhdanov suggested initiating a worldwide program of smallpox eradication at the 9^th^ World Health Assembly (WHA). The corresponding resolution implying complete smallpox eradication was adopted at the 7^th^ WHA plenary meeting on June 12, 1958 [1, 2].

This marked the start of an unprecedented international program of global smallpox eradication under the aegis of the World Health Organization (WHO). The Soviet Union not only played a key role in initiating the eradication program, but it was also an important backer at all stages of its implementation in subsequent years. In 1958, the year of its inception, the Soviet Government offered 25 million doses of dry smallpox vaccines to the WHO, which was then delivered to different countries. In 1960, a laboratory for large-scale production of the vaccine, in compliance with the WHO requirements, was organized at the Institute of Viral Preparations (IVP, Moscow). This laboratory subsequently became a center where professionals from different countries came to master smallpox vaccine manufacturing. A total of over 1.5 billion doses of the smallpox vaccine produced in the Soviet Union were used for mass vaccination in 45 countries over 20 years of the international smallpox eradication program. This represents one of the key roles played by the Soviet Union in the global smallpox eradication [2].

The Laboratory for Smallpox Prevention at the Institute of Viral Preparations played an important role in global smallpox eradication and led to the establishment of the International Reference Center for Smallpox. Numerous Soviet experts were trained at the Center before visiting smallpox-endemic countries and received the necessary preparation for practical work.

Thanks to the joint efforts of the world community in anti-epidemic control and mass anti-smallpox vaccination under the Intensified Smallpox Eradication Programme approved by WHO in 1966, the last natural case of smallpox was recorded in Somalia in October, 1977. Based on the statement made by the Global Commission for the Certification of Smallpox Eradication on May 8, 1980, the 33^rd^ WHA declared that people throughout the globe had overcome smallpox. This was the first, and yet only, victory of the world community over a highly dangerous infectious human disease [1-3].

## THE GENOME PROJECT

Once smallpox had been eradicated, the number of laboratories that stored a smallpox virus named variola virus (VARV) was reduced to stave off the risk of its accidental spread. As early as 1981, only four such laboratories remained (in the United States, the Soviet Union, the Republic of South Africa, and the United Kingdom) and their number was reduced to two in 1984. The latter two laboratories, namely, the Institute of Viral Preparations (Moscow, Soviet Union) and Centers for Disease Control and Prevention (CDC, Atlanta, United States) got the status of WHO Collaborating Centers for Smallpox and Other Poxvirus Infections [1].

Despite strict WHO control, these two repositories of live VARV strains were regarded as a source of potential biological threat. Correspondingly, a decision was made at the 4^th^ meeting of the WHO Committee on Orthopoxvirus Infections (Geneva, 1986) to destroy the collections of VARV strains and their genomic DNAs. Taking into account the planned destruction of VARV collections, it was necessary to reliably preserve the genetic material of various VARV isolates in biologically safe form, as an issue of paramount importance for future research. In order to preserve information about this unique virus, the WHO Advisory Board deemed it necessary to sequence the VARV genome [4].

Correspondingly, A.I. Kondrusev, a deputy minister of public health in the Soviet Union, and Yu.T. Kalinin, a deputy minister of medical industry, approved the *National Program for Conservation of Genetic Material of the Russian Collection of Variola Virus Strains *with L.S. Sandakhchiev, director general of the Scientific and Production Association Vector (VECTOR), and O.G. Andzhaparidze, the director of IVP, as scientific supervisors and S.N. Shchelkunov and S.S. Marennikova as principal researchers.

In December 1990, the 5^th^ meeting of the WHO Committee on Orthopoxvirus Infections approved the national programs for research into the VARV genome proposed by Russia (VECTOR, Koltsovo, Novosibirsk region, and IVP, Moscow) and the United States (CDC, Atlanta, Georgia, and Institute for Genomic Research, Gaithersburg, Maryland). In May 1991, the WHO Commission inspected the VECTOR’s laboratory headed by S.N. Shchelkunov and officially approved the cloning of VARV DNA fragments and their sequencing.

The species specific name of VARV is *Variola virus. *Two subspecies are commonly distinguished: *V. major, *causing the disease with a mortality rate of 5–40% and *V. minor, *with a lethal outcome of less than 2% [1]. VARV is a member of the genus *Orthopoxvirus* belonging to the family Poxviridae. This genus also includes the zoonotic species *Monkeypox virus* (MPXV), *Cowpox virus* (CPXV), *Vaccinia virus* (VACV), *Buffalopox virus* (BPXV, a subspecies of VACV), and *Camelpox virus* (CMLV), all able to infect humans [2-4]. Orthopoxviruses are closely related in their antigenic and immunological characteristics and provide cross-protection when they infect humans or animals [1].

By mid-1992, Russian scientists were first to successfully complete the genome sequencing of a highly virulent VARV *major *strain isolated in India in 1967 during a smallpox outbreak with a mortality rate of 31%, perform a computer analysis of the sequencing data [13-16], and compare them to the then recently published genome sequence of VACV [17, 18]. The results of that work were for the first time reported as an oral presentation at the opening the 9th International Conference on Poxviruses and Iridoviruses [19]. One year later, an American team completed the sequencing and analysis of the whole genome of another highly virulent VARV *major *strain, Bangladesh-1975, isolated during a smallpox outbreak with a mortality rate of 18.5% [20]. Subsequent comparison of the genomes of these strains revealed that they were highly conserved [21, 22].

It was decided at the 6^th^ Meeting of the WHO Committee on Orthopoxvirus Infections (September 1994, Geneva, Switzerland) that the VARV DNA stocks should be stored in two international repositories, namely, VECTOR (by that time with the status of State Research Center of Virology and Biotechnology) and CDC (United States).

The complete genome of a low virulent VARV *minor* strain, Garcia-1966, was sequenced and analyzed (*table*) by collaborating teams from VECTOR and the CDC [23].

**Table:** The first sequenced orthopoxvirus genomes

Species	Strain	Genome size, bp	Number of potential genes	Organization which made sequencing	Year of sequencing
Vaccinia virus	Copenhagen	191636	198	Virogenetics, USA	1990
Variola major virus	India-1967	185578	199	SRC VB VECTOR, Russia	1992
Variola major virus	Bangladesh-1975	186103	196	CDC, USA	1993
Variola minor virus	Garcia-1966	186986	206	SRC VB VECTOR, Russia; CDC, USA	1995
Cowpox virus	GRI-90	223666	212	SRC VB VECTOR, Russia	1997
Vaccinia virus	Ankara	177923	157	Biomedical Research Center, Austria	1998
Monkeypox virus	Zaire-96-I-16	196858	191	SRC VB VECTOR, Russia	2001
Cowpox virus	Brighton Red	224499	218	Duke University Medical Center, USA	2002
Vaccinia virus	WR	194711	206	CDC, USA	2003

Taking into account the potential threat related to manipulations of live VARV in Moscow, the collection of VARV strains was transferred from the Institute of Viral Preparations to VECTOR (Koltsovo, Novosibirsk region) in September, 1994, under a joint order from the Russian Ministry of Health and Medical Industry, the Ministry of Science, the State Committee on Sanitary and Epidemiology Surveillance, and the Academy of Medical Sciences.

The WHO officially registered the organization of the WHO Collaborating Center for Orthopoxvirus Diagnosis and Repository for Variola Virus Strains and DNA at SRC VB VECTOR in June 1997, after a WHO Commission, in 1995, had inspected the laboratory facilities providing the highest degree of physical safety intended for this purpose. The right of VECTOR to keep the repository of VARV strains and their genomic DNAs was officially approved by WHA Resolution no. 49.01 and confirmed by later Resolutions nos. 52.10, 55.15, and 60.1.

The WHO Advisory Committee on Variola Virus Research was organized in 1999 to oversee manipulations with VARV and holds annual meetings for all experts involved in relevant studies and in the development of diagnostic, prevention, and therapeutic tools for smallpox and other human orthopoxvirus infections.

To gain insight into the evolutionary interactions of different orthopoxvirus species, it became necessary to compare their genomes. The VECTOR’s team was the first to sequence the genome DNAs of CPXV [24] and MPXV [25, 26] isolated from sick individuals (*Table*). The analysis of the complete VARV, MPXV, CPXV, VACV, and CMLV genomes made it possible to establish that the CPXV DNA is not only the longest among the studied orthopoxviruses, but also contains all the genetic elements characteristic of the remaining orthopoxvirus species [24, 27-31]. VARV, MPXV, and VACV can be regarded as CPXV variants with deletions, rearrangements, and point mutations specific to each individual species. This suggested to us that a CPXV-like virus is the ancestor of all extant orthopoxvirus species pathogenic for humans [24, 26, 32].

The accumulated data laid the basis for the pioneering comparative analysis of the genomic strategies utilized by all orthopoxvirus species pathogenic for humans, the first phylogenetic studies of this virus group, and the discovery of their evolutionary relationships. However, that data has not yet allowed us to date the molecular evolution of orthopoxviruses and, in particular, VARV [26, 33-36].

The issue of dating the VARV molecular evolution essentially shifted when the VECTOR and CDC teams designed a method allowing one to detect genetic differences between VARV strains. The method utilizes complete VARV genomes and includes long-distance polymerase chain reaction (LPCR) of overlapping genomic segments of the virus’ DNA (with a length of 10 kbp and longer) with subsequent hydrolysis of the synthesized amplicons by frequently cutting restriction endonucleases, electrophoresis, and a computer-aided analysis of restriction fragment length polymorphism (RFLP). This relatively simple approach (the LPCR–RFLP assay), which is close to sequencing in its information content (analyzing the positions of over 300 recognition sites for several restriction endonucleases in a virus DNA sequence), has for the first time made it possible to discover detailed differences between the genomes of 63 VARV strains from the Russian and U.S. collections isolated in several geographic regions and in different years. Phylogenetic analysis of the RFLP data for viral DNAs allowed us to pioneer the discovery that the West African and South American VARV strains form a separate subtype (clade) that significantly differ in their genome organization from the remaining, studied geographic variants of VARV [37]. It is essential here that the West African and South American VARV strains within the discovered subtype form two distinct phylogenetic groups (subclades), which suggests their independent evolution over a certain time period. The results of this analysis and the historical facts that VARV had been several times imported from West Africa to South America in the 16th–18th centuries through slaves allowed us to quantitatively estimate the rate of poxvirus evolution for the first time [38].

Sequencing of the complete genomes of a large set of VARV strains isolated in different years and geographic regions [39], as well as extended genome segments of several additional VARV strains [40], made it possible to more precisely date the key events in VARV evolution [41, 42].

## POSSIBLE SMALLPOX REEMERGENCE

Taking into account the fact that smallpox vaccination in several cases had adverse side effects, the WHO recommended ceasing vaccination after 1980 in all countries. The result of this decision was that mankind lost its collective immunity not only to smallpox, but also to other zoonotic orthopoxvirus infections. The ever more frequently recorded human cases of zoonotic orthopoxvirus infections force us to revisit the problem of possible smallpox reemergence resulting from a natural evolution of these viruses [32, 43].

An important feature of VARV is its ability to infect only humans and the absence of a natural reservoir (a sensitive animal species). One should keep in mind that VARV infection of a human can in many cases (up to 40% and more) result in a lethal outcome [1-4].

MPXV causes a human disease that resembles smallpox in its clinical manifestations and also may result in a lethal outcome in up to 10% of cases. The major difference between the human monkeypox and smallpox consists in a low human-to-human transmission efficiency of the former, which so far has prevented the development of local monkeypox outbreaks into epidemics [44]. However, some recent data demonstrate that the efficiency of MPXV spread in human populations is growing [45, 46], which should cause concern in both the medical communities in Central and West Africa and at the WHO.

MPXV in the long-term absence of population-scale vaccination and an increased rate of human monkeypox cases can well acquire the ability to spread from human to human more efficiently, as is characteristic of VARV. If this happens, mankind will face a much more complex problem as compared to smallpox eradication. First and foremost, this will have to do with the fact that MPXV, unlike VARV, has a natural reservoir; namely, an abundant African rodent species [32].

Other zoonotic orthopoxvirus species typically cause sporadic human infections (small-scale outbreaks) with a benign outcome in most cases [6, 9, 12]. However, it is known that human infection with CPXV can lead to a generalized disease resembling smallpox with a lethal outcome in immunodeficient individuals [47, 48].

As mentioned above, the comparative analysis of the genomes of VARV and the zoonotic orthopoxviruses pathogenic for humans has shown that CPXV has the largest genome containing all the genes characteristic of the remaining orthopoxvirus species. Part of the genes in other orthopoxviruses is broken or deleted, and individual orthopoxviruses have species-specific differences in their set of retained genes. These data support the concept of reductive evolution of orthopoxviruses, according to which the loss of genes plays an important role in the evolutionary adaptation of an ancestral virus to a certain host species, as well as in the emergence of new virus species [49, 50]. VARV, the virus most pathogenic to humans, possess the smallest genome among all orthopoxviruses. This indicates a possibility that a VARV-like virus can evolve from extant zoonotic orthopoxviruses with a longer genome as a result of natural evolution [32, 42].

An analysis of available archive data on smallpox epidemics, the history of ancient civilizations, and the most recent data on the evolutionary relationship between orthopoxviruses has allowed us to hypothesize that VARV could have repeatedly reemerged via evolutionary changes in a zoonotic ancestor virus and then disappeared because of an insufficient population size of isolated ancient civilizations [43]. Only the historically latest smallpox pandemic raged for a long time and was contained and stopped in the 20^th^ century thanks to the joint efforts of medical professionals and scientists from many countries under the aegis of the WHO.

Therefore, the reemergence of smallpox or a similar human disease in the future in the course of a natural evolution of currently existing zoonotic orthopoxviruses is not impossible. Correspondingly, it is of utmost importance to develop and widely adopt state-of-the-art methods for an efficient and rapid species-specific diagnosis of all orthopoxvirus species pathogenic for humans, including VARV. It is also important to develop new safe methods for the prevention and therapy of human orthopoxvirus infections.

## SPECIES-SPECIFIC DNA DIAGNOSTICS OF ORTHOPOXVIRUSES

The characteristics of orthopoxvirus infections are similar external manifestations, including skin lesions; however, experience has shown that clinical diagnosis of these diseases is frequently erroneous [3, 4].

The advent of the polymerase chain reaction technique has resulted in cutting-edge methods that allow for the detection and identification of trace amounts of microorganisms in assayed samples with a high specificity and over a short time [51]. Moreover, and most importantly, these methods require no manipulations with live specific pathogens, including VARV and MPXV.

In the case of orthopoxviruses pathogenic for humans, test kits that provide genus-specific DNA identification for an assayed virus with concurrent species-specific differentiation are a priority. The VECTOR team was the first to elaborate such methods based on classical multiplex PCR [52, 53] and multiplex real-time PCR [54-58].

The method that utilizes oligonucleotide microarrays is also based on PCR; in the assay, the synthesized DNA amplicons are identified by hybridization with specific oligonucleotides immobilized on a support in a particular order. The DNA preparations to be assayed in hybridization are fluorescently labeled. After hybridization and washing, the microarray is analyzed with the help of a laser scanner and the recorded fluorescence data for each cell of the micromatrix are processed using specialized software. Similar to classical PCR, this method can allow one to detect trace amounts of the analyte in a specimen. One of the important advantages of oligonucleotide microarrays is the possibility to simultaneously analyze a multitude of genetic loci, thereby considerably increasing the reliability of the method [4].

Different variants of diagnostic oligonucleotide microarrays have been designed for species-specific diagnosis of orthopoxviruses [59-62].

The development of next-generation sequencing technologies would makes it possible to obtain the complete genome nucleotide sequence of a research subject in short order. Genome-wide sequencing of isolated viruses in the case of unusual orthopoxvirus infections is an ever more frequent situation [63, 64]. These studies demonstrate that laboratory diagnostic techniques for orthopoxvirus infections, as well as epidemiological surveillance, need further upgrades. Naturally circulating zoonotic orthopoxviruses pathogenic for humans require a comprehensive study and monitoring for the emergence of new species that can potentially lead to the emergence of new orthopoxvirus variants highly pathogenic for humans while routine smallpox immunization is absent.

## MODERN ANTI-SMALLPOX VACCINES

The first-generation smallpox vaccine was a VACV preparation produced by propagating the virus on calf (or other animal) skin. Today, VARV vaccine strains are produced in mammalian cell cultures and are referred to as second-generation smallpox vaccines [65]. Although vaccine production in cell cultures meets current standards, second-generation smallpox vaccines, similar to first-generation ones, can cause adverse side effects and, thus, are of limited use [66].

Third-generation attenuated smallpox vaccines are produced via multiple passaging of a VACV strain in the cell culture of a heterologous host. For example, the best studied third-generation vaccine, MVA, is produced by multiple passages of the VACV strain Ankara in a chick fibroblast culture. The MVA strain genome has accumulated numerous mutations and long deletions that distinguish it from the initial VACV strain. MVA is unable to replicate in most mammalian cells, including human cells [67].

The vaccine based on the VACV strain MVA (Imvanex/Imvamune) has undergone numerous clinical trials, including studies in subjects with atopic dermatitis and HIV [68-70]. This vaccine is shown to induce an antibody profile that is similar to that induced by the conventional first-generation vaccine and to protect various laboratory animals against zoonotic orthopoxviruses [71-73]. Imvanex/Imvamune has been licensed in European countries, Canada, and the United States. First and foremost, this vaccine is intended for primary vaccination of subjects with contraindications for using first- and second-generation smallpox vaccines.

Another third-generation smallpox vaccine, LC16m8, licensed in Japan, was produced from VACV strain Lister via multiple passages in a primary rabbit kidney cell culture at a decreased temperature (30°C). Clinical studies have demonstrated a considerable reduction in the number of adverse side effects compared to the conventional Lister-based vaccine. The resulting attenuation of this vaccine strain is mainly due to a mutation (single nucleotide deletion) in the *B5R *gene that encodes a protein essential for extracellular enveloped virion formation [74, 75]. The protective efficacy of LC16m8 in animal model experiments is comparable to that of the parental strain Lister [76, 77].

A new approach to the production of fourth-generation attenuated smallpox vaccines consists in genetic engineering of variants with impaired genes that control the host’s protective response to virus infection, the range of sensitive hosts, etc. by introducing targeted deletions/insertions. The best studied variant of such VACV is strain NYVAC, with a deleted block of 12 genes and six additional individual damaged genes. The NYVAC strain induces considerably weaker immunity in humans as compared to the classical Lister or Dryvax vaccine, including the inability to induce A27-specific antibodies, which are necessary for efficient neutralization of a VACV infectious form, the intracellular mature virus [78, 79].

In Russia, a highly attenuated VACV variant was produced by successive introduction of targeted deletions/insertions into five individual genes of strain LIVP [80]. Additional targeted deletion introduced into the *A35R *gene yielded another highly immunogenic attenuated strain, VACdelta6 [81], which is currently under preclinical trials as a fourth-generation smallpox vaccine candidate. This vaccine can be used in combination with the smallpox DNA vaccine [82].

## ANTI-SMALLPOX CHEMOTHERAPEUTICS

Chemotherapeutics are no less important in the treatment of human orthopoxvirus infections, and the search for such drugs over the past 20 years has been a success. Since there are no adequate animal models for smallpox, potential anti-smallpox drugs are tested in surrogate smallpox animal models [83]. The inhibitors of orthopoxvirus reproduction were initially screened in cell cultures to further study the compounds with high *in vitro *antiviral activity using animal models, first and foremost, intranasal or aerosol infection of mice with CPXV and monkeys with MPXV [84, 85]. Rabbits infected with the rabbitpox virus (RPXV) and ground squirrels infected with MPXV have recently been actively used [86-88]. However, none of the surrogate animal models of orthopoxvirus infection precisely reproduces human smallpox. Correspondingly, the candidate compounds are examined in parallel, using several animal models.

Cidofovir, an antiviral nucleotide analog (brand name Vistide) officially approved for clinical use against cytomegalovirus retinitis and acting as an inhibitor of virus DNA polymerase, was the first compound intensively studied as an anti-orthopoxvirus drug [83]. Cidofovir proved efficient against orthopoxvirus infections in different animal models; however, its essential shortcomings are poor water solubility and mandatory intravenous administration. Correspondingly, a lipid cidofovir conjugate, CMX001 (Brincidofovir), has been synthesized [86, 89]. It is a broad-spectrum drug with pronounced anti-orthopoxvirus activity and is also administrable in tablet form.

ST-246, a compound that blocks the final stage in the assembly of intracellular enveloped virions and prevents the release of the virus from an infected cell [83, 90], is of the greatest interest. ST-246 was identified by screening a library comprising over 350 thousand unique compounds for antiviral activity. ST-246 (Tecovirimat) has shown low toxicity and high antiviral efficacy in mice infected with ectromelia virus, VACV, and CPXV; rabbits infected with RPXV and ground squirrels infected with MPXV; and monkeys infected with MPXV or VARV [90-92]. This compound is currently undergoing clinical trials. NIOCH-14, an analog of ST-246, also showed high activity in different animal models of orthopoxvirus infections [93].

The search for new anti-orthopoxvirus chemotherapeutic agents with other molecular targets is in progress [90, 94].

## CONCLUSIONS

The analysis of the genome organization of orthopoxviruses pathogenic for humans and their patterns of evolution suggest the fundamental possibility that smallpox or a similar human disease can emerge in the future via a natural evolution of extant zoonotic orthopoxviruses. Cessation of anti-smallpox vaccination and the resulting loss of collective population immunity not only to smallpox, but also to other orthopoxvirus infections creates conditions that promote the spread of zoonotic orthopoxviruses among people, thereby potentially enhancing the selection of virus variants highly pathogenic for humans and epidemically dangerous. However, the situation today does not look irredeemable and radically differs from the events far past, when man had no control over infections. Today, most outbreaks of orthopoxvirus infections in domestic animals and humans are registered and investigated; in addition, the efficient international system for clinical sampling and identification of infectious agents has been validated and anti-epidemic activities and protocols for mass vaccination were developed during the implementation of the global smallpox eradication program [1].

The recent efforts at the WHO are directed towards developing state-of-the-art methods for rapid VARV identification and designing next-generation safe anti-smallpox vaccines and chemotherapeutic agents against VARV and other orthopoxviruses [94].

The studied vaccines and chemotherapeutics are not strictly species-specific with respect to orthopoxviruses pathogenic for humans and, thus, are applicable to outbreaks caused by any orthopoxvirus species. Taking into account the above succinct information, it results that diagnostic methods should be focused on rapid identification not only of VARV, but also of MPXV, CPXV, VACV, and CMLV [32]. The recent increase in the number of outbreaks of orthopoxvirus infections in animals and humans and the potential danger they pose demonstrate the importance of constant monitoring of these infections all over the world aimed at insuring against the development of small outbreaks into epidemics and, thus, decreasing the risk of an emergence of a new orthopoxvirus highly pathogenic for humans.

Phenomenal advance in synthetic biology has made it possible to *de novo *synthesize the complete horsepox virus genome and obtain a live virus [95]. This suggests that any orthopoxvirus, including VARV, can be recreated in a laboratory. That is why the development and wide clinical application of the most advanced methods for the diagnosis, prevention, and therapy of orthopoxvirus infections pose a vital challenge.

## Abbreviations

BPXV: buffalopox virus
CMLV: camelpox virus
CPXV: cowpox virus
MPXV: monkeypox virus
PCR: polymerase chain reaction
RPXV: rabbitpox virus
VACV: vaccinia virus
VARV: variola virus
WHA: World Health Assembly
WHO: World Health Organization
